# Epidemiology and Perception of Acne Among Adolescents in Jos, Nigeria: Cross-Sectional School-Based Study

**DOI:** 10.2196/44441

**Published:** 2023-07-12

**Authors:** Ruth Adah, Hope Yusufu, Queen-Amina Vivian Otene

**Affiliations:** 1 Department of Paediatric Jos University Teaching Hospital Jos Nigeria; 2 Faculty of Medicine College of Health Science University of Jos Jos Nigeria

**Keywords:** prevalence, predictors, misconception, perception, groundnuts, pimples, teenagers, acne, dermatology, Nigeria, acne vulgaris, comedone, papule, pustule, nodule, cyst, blackhead, whitehead

## Abstract

**Background:**

Adolescents who make up a vast majority of the secondary school population are at a stage at which they are largely affected by acne. This condition, which is widely visible and easily recognized by peers, has numerous misperceptions surrounding it, which may influence attitudes toward people affected by it. There is a paucity of information on the prevalence of acne and how adolescents in Jos, Nigeria, view the condition.

**Objective:**

This study aimed to determine the prevalence of acne, perceived risk factors, and the accuracy of self-report among adolescents in Jos, Nigeria. The study also sought to understand perceptions surrounding acne in this age group.

**Methods:**

This descriptive cross-sectional study was conducted among adolescents attending private and public secondary schools in Jos, Nigeria. In total, 482 students were recruited through a multistaged stratified random sampling method. A self-administered semistructured questionnaire was used to collect information on history of acne, perceptions of causes, and the attitude toward those who have the condition. All participants were examined for the presence of acne. Univariate, bivariate, and multivariate analysis were conducted using SPSS (version 26; IBM Corp).

**Results:**

The self-reported prevalence of acne was 44% and that upon clinical examination was 55%. Self-report showed a moderate degree of agreement with clinical diagnosis (Cohen κ=57.3%; *P*<.001). Predictive factors for the presence of acne in general were age of ≥15 years (odds ratio [OR] 1.79, 95% CI 1.12-2.87; *P*=.02), being in a private school (OR 2.17, 95% CI 1.38-3.42; *P*=.001), and being in a senior secondary class (OR 2.14, 95% CI 1.32-3.47; *P*=.002). The female gender (OR 3.03, 95% CI 1.64-5.61; *P*=.001) and religion (OR 3.24, 95% CI 1.27-8.24; *P*=.02) were predictive for acne only among adolescents aged <15 years, while a positive family history was predictive in those aged ≥15 years (OR 2.04, 95% CI 1.15-3.61; *P*=.02). A distinct perception and attitude pattern surrounding acne was observed, as a significant proportion (84/131, 64.1% vs 47/131, 35.9%; *P*=.02) of those who related acne to a biological phenomenon had acne themselves; however, the belief that acne is caused by skin lightening practices was significantly more common in those without acne (19/28, 67.9%) than in those with acne (9/28, 32.1%; *P*=.01). One-fourth of the adolescents (n=122, 25.3%) had no idea of the possible causes of acne.

**Conclusions:**

Though acne is a prevalent skin condition among Nigerian adolescents, many misperceptions and unfavorable attitudes surround acne and persons affected by the condition. Our findings have revealed the need to work with the school health program to educate the general adolescent population about acne, to refer and manage teenagers with acne.

## Introduction

Acne vulgaris is a disease of the pilosebaceous units of the skin of the face, neck, chest, shoulders, and back. The condition typically starts at puberty and is induced by abnormal follicular keratinization, excessive sebum production, *Propionibacterium acnes* colonization, and localized inflammation. In addition to the typical lesions of comedones, papules, pustules, nodules, and cysts, there may be scarring and postinflammatory hyperpigmentation [1,2]. According to the Global Burden of Disease study [2], the estimated global prevalence of acne in 2010 was 9.38%, and acne ranked as the eighth most prevalent skin condition worldwide.

Few conditions exist where there is significant prevalence of acne across countries and cultural groups. Reported prevalence varies in accordance with age groups and is influenced by the different methods used in studies. Identification of acne by experts through clinical examination in certain studies have revealed a prevalence almost equivalent to the total number of adolescents examined [3-5]. Studies based on adolescent self-report have also reported substantial prevalence rates (49.8%-83.4%) [6-8]. Self-report by affected individuals has shown some validity as fair to good agreement with experts’ diagnosis has been demonstrated, albeit insufficient for both treatment and research purposes [9-11].

The etiology of acne is multifactorial, and diet has frequently been implicated as a risk factor in acne occurrence and severity. High-glycemic-index foods, including those with high carbohydrate and sugar content, combined with low intake of vegetables and high intake of dairy products have been associated with the occurrence or severity of acne [12,13]. These factors are known to be individualized and influenced by genetics; thus, family history has also been identified as a predisposing factor [1]; other commonly documented factors are heat and humidity, overweight, use of skin and hair care products such as pomades [14,15]. Furthermore, there are many acne-related beliefs held with little supporting evidence, which influence health-seeking behaviors and attitudes toward persons with acne [8,16]. Inadequate awareness of acne has also been linked to stigma, discrimination, low self-esteem, depression, and suicidal thoughts in affected adolescents who are still undergoing physical and psychological maturation. Thus, taking into account adolescents' knowledge of acne is useful in developing approaches to health education and management [17-19].

Studies have reported that the prevalence of acne in adolescents and youths in Nigeria is between 30% and 90.7%, with documented associated factors possibly unique to those regions where the studies were conducted—mostly Southern Nigeria [4,8,12,20]. For instance, Jos, seated on a plateau that has cooler weather with food items cultivated and consumed that are unique to the state, may have a distinct epidemiology of acne among its adolescents. This study, therefore, aimed at ascertaining the prevalence of acne among adolescents in Jos, Nigeria, associated factors, and the reliability of self-report when compared to clinical diagnosis. An additional goal of the study was to document adolescents' perceptions about the causes and attitudes toward persons with acne.

## Methods

### Overview

Jos is the capital of state of Plateau in North-Central Nigeria, with a significantly cooler temperature than that in other states in Nigeria. It is the center of cultivating vegetables and tubers, which are distributed to other parts of the country. Many settlements in Jos are characterized by persons of similar religion or tribe with their distinctive cultures. Schools are, therefore, influenced by this pattern, such that their populations reflect that of the host community. Furthermore, access to schools in the country is largely based on socioeconomic status as students from poorer homes attend public schools with lower funding while children from families of upper socioeconomic class, who can afford private schools, attend private schools [21].

This cross-sectional study was conducted among adolescents at 4 secondary schools in 2 Local Government Areas (LGAs) of Jos Metropolis within a 4-week period, between September and October 2022. Multistage random sampling was used to select 482 students in the schools in Jos North and Jos South LGAs (1 private and public secondary school in each LGA). The eligibility criteria for schools were that they were coeducational day schools with all 6 class years—the first 3 years termed as junior secondary and the last 3 as senior secondary. Participants were chosen from a sampling frame of each school, which included every class throughout the school using a predetermined sampling interval.

Information regarding demographic characteristics, first-degree family history of acne, perceptions regarding the causes of acne, and the impression that study participants have of others with acne were collected using self-administered questionnaires. All participants had their anthropometric measures (weight and height for BMI calculation) taken and examined privately for acne by a pediatric dermatologist. Participants with a BMI of <18.5 were classified as undernourished; those with a BMI of 18.5 to 24.9 as normal, and those with a BMI of ≥25 as overnourished. All forms of acne (inflammatory and noninflammatory) were recorded as “Acne present.” Data collected were entered into SPSS Statistics for Windows (version 26.0; IBM Corp).

Data were tabulated as frequency and percentage values, while numeric variables were presented as means. The Pearson chi-square test and Fisher exact test were used to analyze the association between categorical groups, and multinomial logistic regression was performed to explore the relationship between the predictor variables and the presence of acne. Odds ratios (ORs) with 95% CIs were used to state the measure of relationship between variables. A *P* value of <.05 was determined to be the level of statistical significance.

### Ethical Considerations

The study was approved by the Research and Ethical Committee of the Jos University Teaching Hospital, Jos (JUTH/DCS/IREC/127/XXX1/2750). Permission was sought from each school, and written informed consent was obtained from the guardians or parents of the students prior to their involvement. Participation was voluntary, and each student could withdraw from the study at any time.

## Results

### Respondent Demographic Characteristics

Of 482 students selected and provided consent forms for their parents or guardians, 14 of them refused to consent, did not have capacity to fully understand and fill in the questionnaire, or were absent, so they were replaced by the next student on the list whose parents or guardians had provided consent.

The age range of the participants (9-21, mean 14.6, SD 2.2 years) was wider than expected in Nigerian secondary schools (11-17 years). There were more female (292/482, 60.6%) than male students. The general ratio of male and female students was 1:1.5; however, the male:female ratio in public schools (1:2.1) was higher than that in private schools (1:1.2). Students’ BMI ranged from 12.9 to 29.9 (mean 19.5, SD 3.1) with only 24 of 482 (5.0%) being overnourished (having overweight and obesity) and 189 (39.2%) being undernourished (Table 1).

**Table 1:** Frequency distribution of the characteristics of the study participants (N=482).

Characteristics		Participants, n (%)
**Gender**		
	Female	292 (60.6)
	Male	190 (39.4)
**Age group (years)**		
	<15	228 (47.3)
	≥15	254 (52.7)
**Religion**		
	Christianity	377 (78.2)
	Islam	105 (21.8)
**School**		
	Private	242 (50.2)
	Public	240 (49.8)
**Tribe**		
	Other tribes	153 (31.7)
	Plateau indigenes	329 (68.3)
**Class**		
	Junior secondary class	257 (53.3)
	Senior secondary class	225 (46.7)
**Nutritional status**		
	Underweight or undernourished	189 (39.2)
	Normal	269 (55.8)
	Overnourished (having overweight and obesity)	24 (5)
**Family history of acne**		
	No	180 (37.3)
	Yes	302 (62.7)

### Acne Prevalence by Self-Report and Examination

Only over one-third (n=188, 39%) of respondents said that they experienced acne in the past, while less than half (n=213, 44.2%) of the respondents self-reported having acne at the time of the study, while on clinical examination, more than half (n=265, 55%) of them were found to actually have acne.

There was an 88% chance of adolescents properly stating when acne was absent because only 12% of self-reported cases of acne were false positive (specificity). The sensitivity of self-report compared to that of clinical diagnosis was 70.6%, with 78 (29.4%) participants who self-reported having no acne at the time of the study actually deemed as having acne upon clinical diagnosis (false negative). The negative predictive value was 71%, and the positive predictive value was 87.8%.

On assessing the presence of acne, a weighted κ of 57.3% (*P<*.001) indicates moderate consistency between the adolescents’ self-report and examination by a pediatric dermatologist. Table 2 summarizes the prevalence of acne by self-report and on clinical examination, and Table 3 compares the accuracy of adolescents’ self-reports of acne to their clinical diagnoses.

**Table 2:** Prevalence of acne by self-report and clinical examination (N=482).

Presence of acne		Participants, n (%)
**Do you currently have acne (self-report)?**		
	Yes	213 (44.2)
	No	269 (55.8)
**History of acne in the past (irrespective of current status)**		
	Yes	188 (39)
	No	294 (61)
**Having acne on clinical examination**		
	Yes	265 (55)
	No	217 (45)

**Table 3:** Comparison of the accuracy of adolescents’ self-report of acne with that of their clinical diagnosis (N=482).

Self-report of acne	Acne by examination, n (%)	
	Present	Absent
Yes	187 (70.6)	26 (12)
No	78 (29.4)	191 (88)

### Characteristics Associated With the Presence of Acne

Multinomial logistic regression was used to analyze the relationship between certain characteristics and the presence of acne (Table 4). Five variables were found to be predictive of the presence of acne: age of ≥15 years (OR 1.78, 95% CI 1.11-2.86; *P*=.02), female gender (OR 1.80, 95% CI 1.19-2.71; *P*=.01), identifying as Christian (OR 2.1, 95% CI 1.10-4.01; *P*=.02), studying at a private school (OR 2.17, 95% CI 1.38-3.42; *P*=.001), and being in a senior secondary class (OR 2.14, 95% CI 1.32-3.47; *P*=.002).

**Table 4:** Multinomial logistic regression of factors associated with the presence of acne.

Factors		Adjusted odds ratio (95% CI)	*P* value	
**Age group (years)**				.02^a^
	<15	1 (reference)		
	≥15	1.78 (1.11-2.86)		
**Gender**				.01^a^
	Male	1 (reference)		
	Female	1.80 (1.19-2.71)		
**Religion**				.02^a^
	Muslim	1 (reference)		
	Christian	2.10 (1.10-4.01)		
**Tribe**				.55
	Plateau Indigenous	1 (reference)		
	Others	1.19 (0.68-2.08)		
**School type**				.001^a^
	Public	1 (reference)		
	Private	2.17 (1.38-3.42)		
**Class**				.002^a^
	Junior secondary	1 (reference)		
	Senior secondary	2.14 (1.32-3.47)		
**Known family history**				.09
	Yes	1 (reference)		
	No	0.7 (0.47-1.11)		
**BMI status**				
	Underweight	1 (reference)	N/A^b^	
	Normal	2.15 (0.57-6.13)	.15	
	Overweight	1.31 (0.48-3.56)	.60	

^a^Significant at *P*<.05.

^b^N/A: not applicable.

When separated into 2 major groups based on age, of 95 of 228 (41.7%) adolescents younger than 15 years had acne, and 170 of 254 (66.9%) adolescents aged 15 years and older were found to have acne. School type and class remained predictive of the presence of acne in both age groups. The female gender (OR 3.03, 95% CI 1.64-5.61; *P*=.001) and religion (OR 3.24, 95% CI 1.27-8.24; *P*=.02) were predictive for acne only in adolescents aged <15 years, while positive family history was predictive in those aged ≥15 years (OR 2.04, 95% CI 1.15-3.61; *P*=.02; Table 5).

**Table 5:** Factors associated with the presence of acne in different age groups.

			Participants aged <15 years				Participants aged ≥15 years		
			Odds ratio (95% CI)		*P* value		Odds ratio (95% CI)		*P* value
**Gender**					<.001^a^				.68
	Male	1 (reference)				1 (reference)			
	Female	3.03 (1.64-5.61)				1.13 (0.62-2.06)			
**Religion**					.02^a^				.39
	Muslim	1 (reference)				1 (reference)			
	Christian	3.24 (1.27-8.24)				1.52 (0.58-4.00)			
**Tribe**					.32				.85
	Plateau Indigenous	1 (reference)				1 (reference)			
	Others	1.46 (0.69-3.06)				1.09 (0.45-2.63)			
**School**					.03^a^				.01^a^
	Public	1 (reference)				1 (reference)			
	private	2.08 (1.08-4.01)				2.50 (1.28-4.85)			
**Class**					.03^a^				.02^a^
	Junior	1 (reference)				1 (reference)			
	Senior	2.42 (1.11-5.28)				2.06 (1.1-3.88)			
**Known Family History**					.84				.02^a^
	Yes	1 (reference)				1 (reference)			
	No	1.06 (0.59-1.93)				2.04 (1.15-3.61)			
**BMI**									
	Underweight	1 (reference)		N/A^b^		1 (reference)		N/A	
	Normal	1.44 (0.79 -2.63)		.24		3.30 (0.88-12.36)		.08	
	Over-nourished	1.42 (0.18-11.06)		.74		1.58 (0.48-5.20)		.45	

^a^Significant at *P*<.05.

^b^N/A: not applicable.

### Perception and Attitudes Related to Acne

When asked what they thought causes acne, a large proportion of respondents (122/482, 25.3%) reported having no idea about the causes of acne. Factors believed to be risk factors of acne, as cited by the study participants, were eating groundnuts or peanuts (n=132, 27.4%), eating other dietary items (n=46, 9.5%), acne being a natural or biological phenomenon (n=131, 27.2%), using skin care products or cosmetics (n=45, 9.3%), using skin lightening products (n=29, 6%), health and hygiene (n=66, 13.7%), weather (n=29, 6%), and others (n=17, 3.5%).

Interestingly, participants with acne had significantly different perceptions surrounding acne from those without acne. A larger proportion of adolescents (84/131, 64.1%; *P*=.02) perceived that acne was due to a biological phenomenon. Among those who perceived acne as being caused by skin lightening practices, 19 of 28 (67.9%, *P*=.01) did not have acne. When asked to express their opinions of acne in individuals in 1 word, respondents chose the following: unattractive, not bad, ugly, matured, developed, unhygienic or not clean, unhealthy, pitiable, awful, nasty, overweight, and unhappy people. When these perceptions were grouped together as favorable or unfavorable attitudes, participants without acne had significantly less favorable attitudes toward those with acne than did those with the condition themselves (27.3% vs 72.7%; *P*=.04). In addition, 132 (27.4%) participants also believed acne to be contagious (Table 6).

**Table 6:** Perceptions of the causes of acne and attitudes toward individuals with acne.

Perceived causes of acne or pimples		Acne clinical on examination		Chi-square (*df*)		*P* value	
		Present	Absent				
**Eating groundnuts or peanuts**					2.3 (1)		.13
	Mentioned	80 (60.6)	52 (39.4)				
	Not mentioned	185 (52.9)	165 (47.1)				
**Consuming other foods (greasy or fatty foods, milk, eggs, beans, sugary items, and soft drinks)**					0.7 (1)		.39
	Mentioned	28 (80.9)	18 (39.1)				
	Not mentioned	237 (54.4)	199 (45.6)				
**Biological or natural phenomenon (maturity, period or premenstrual, or familial)**					6.1 (1)		.02^a^
	Mentioned	84 (64.1)	47 (35.9)				
	Not mentioned	181 (51.6)	175 (48.4)				
**Skin-related (using skin care products, cosmetics, and skin type)**					0.5 (1)		.82
	Mentioned	24 (53.3)	21 (46.7)				
	Not mentioned	241 (55.1)	196 (44.9)				
**Skin lightening practices or bleaching**					6.3 (1)		.01^a^
	Mentioned	9 (32.1)	19 (67.9)				
	Not Mentioned	256 (56.4)	198 (43.6)				
**Health and hygiene (dirt, poor hygiene, or disease)**					3.2 (1)		.07
	Mentioned	28 (44.4)	35 (55.6)				
	Not mentioned	237 (90.2)	182 (43.4)				
**Environmental factors (weather and heat)**					0.17 (1)		.68
	Mentioned	17 (58.6)	12 (41.4)				
	Not mentioned	248 (54.7)	208 (45.3)				
**Others (sex, drugs, hormone intake, insects bites, smoking, face-touching, shaving, stress, cloths, bed-wetting, and chalk-dust)**					0.34 (1)		.56
	Mentioned	14 (60.9)	9 (3.7)				
	Not mentioned	251 (54.7)	209 (45.3)				
**Perception of persons with acne (single response)**					6.5 (2)		.04^a^
	Favorable	32 (72.7)	12 (27.3)				
	Unfavorable	76 (51.4)	72 (48.6)				
	No response	157 (54.1)	133 (45.9)				

^a^Significant at *P*<.05.

## Discussion

### Principal Findings

This is one of the few studies conducted in North-Central Nigeria, and, to the best of our knowledge, the only one in Jos to investigate the prevalence of (by self-report and clinical examination) and perceptions surrounding acne in adolescents.

We found that 265 of the 482 (55%) study participants had acne, implying that despite differences in geography, culture, and lifestyle, acne vulgaris is as prevalent among adolescents in Jos as it is in other regions of Nigeria and globally [4-8,20]. The prevalence in Jos is higher than that in Ilorin, another town in North-Central Nigeria, where 40.1% of secondary school students reportedly had acne [20], but lower than that in Kaduna (North West Nigeria), where 90.7% secondary school students reportedly had acne [8]. The huge disparity in prevalence between the Kaduna study and our study could result from the fact that occurrence and severity of acne increase with advancing age and development, and the mean age of our study participants was lower than that of those in the Kaduna study (14.6 vs 16.1 years). Bagatin et al et [3] reported a similar pattern to a greater degree in Brazil where acne was universal in teenagers older than 14 years, such that analysis of associated factors had to be restricted to only those aged 10-13 years.

While expert diagnosis through clinical examination is the gold standard, self-report in our study still revealed a significant prevalence of 44%, which was similar to that reported by Ražnatović Đurović et al [6] (49.8%) among adolescents aged 14-17 years in Montenegro. The self-report rate in our study was lower than that determined through clinical diagnosis (44% vs 55%). A similar pattern was observed in a study among final-year female medical students in Jeddah, Saudi Arabia, when self-evaluation was contrasted with a doctor's examination (83.4% vs 98%) and in Kaduna, Nigeria (59.4% vs 90.7%) [7,8]. Despite the difference between the 2 methods in our study, self-reports agreed fairly well with the expert’s diagnosis with the specificity higher than the sensitivity (88% vs 70.6%). This implies that the accuracy of adolescents’ recognition of when they did not have acne was higher than that when they had acne, which is consistent with that reported in other studies [7,9]. This is expected as our study participants are not trained observers with a capacity for identifying all forms of acne lesions. Also, acne in certain regions, such as the posterior trunk, may not be visible. Still, the condition is so commonplace that 7 of 10 adolescents reported it correctly. In contrast, among Egyptian adolescents, a higher prevalence of acne was determined through self-report than through an experts’ diagnosis (34.7% vs 24.4%) [22]. Higher self-reports of acne and other skin disorders in general have been linked to the availability of resources in health systems, exposure to information, and increased self-awareness, although the experiences of adolescents in our setting may differ [23]. This demonstrates the limitations of self-report and supports the case for dermatologists’ participation in education for and management of prevalent skin conditions of children and adolescents within the school health program [9-11,22].

It was not unexpected to find older adolescents to be almost twice as likely than younger adolescents to have acne, as this condition occurs more frequently with advancing age or maturity [8,11]. Furthermore, older, more mature adolescents are likely to be in higher classes, consequently having an older age (OR 1.78, 95% CI 1.11-2.86; *P*=.02) as well as being in a senior class (OR 2.14, 95% CI 1.32-3.47; *P*=.001) were predictive of the presence of acne [24]. The female gender was only predictive of the presence of acne in younger adolescents (OR 3.03, 95% CI 1.64-5.61; *P*=.001) probably because female individuals in early adolescence attain puberty earlier than male individuals, but by the older adolescent stage, male individuals will have caught up, thus eliminating any gender difference in acne prevalence in older adolescents as seen in this study. This is in agreement with the findings of Anaba et al [25], where a higher acne prevalence was found among female individuals. This, however, differs from studies by Bagatin et al [3] and Yahya et al [8] where prevalence was higher in male individuals. Generally, adolescents in private schools were more likely to have acne (OR 2.17, 95% CI 1.38-3.42; *P*<.001) than those in public schools and it remained consistently so across age groups, as observed by Tayel et al [22] in Egypt and Okoro et al [24] in Southern Nigeria. School type is known to be determined largely by socioeconomic status, which also influences lifestyle, diet, and stress levels and affects acne [22,24].

The relationship between religious practices and acne has scantily been explored, though some studies have suggested that the hijab worn by female students, which covers the scalp and part of the face, increases the likelihood of acne on the forehead and hairline [26]. Our results did not align with this finding, as Christian adolescents had a significantly higher prevalence of acne (OR 2.1, 95% CI 1.10-4.01; *P*=.02) than those of the Islamic faith, although the hijab is the predominant Muslim female attire even in school uniforms. While the exact reason is unknown, there may be underlying genetic and lifestyle-related differences between the 2 religious groups. This highlights the contribution of the frequent face washing during ablution in acne control. Muslims wash the face with water as much as 5 times a day. While water alone may be insufficient in controlling the underlying factors of acne formation, it may remove some surface impurities such as dirt, sweat, and some oil from the surface of skin, preventing pore blockage that is implicated in the etiology of acne [27]. This hypothesis may need further exploration. Familial predisposition of acne has been documented in various studies [1,12]. We, however, found that family history was predictive of acne only in older adolescents but not in younger adolescents (OR 2.04, 95% CI 1.15-3.61; *P*=.02), which could be attributable to possible response bias, as older adolescents are more likely to be aware of their family’s history of acne or be observant of first-degree family members with acne than are younger adolescents. Recent systematic reviews have shown an increased risk for acne among individuals with overweight or obesity in relation to those with a normal BMI or with underweight [1,14]. This pattern was not observed in our study but adolescents who were undernourished consistently had a lower prevalence of acne than those with a normal or overnourished nutritional status, the difference was not significant across both age groups.

Diet featured prominently as a perceived risk factor for acne among our study participants, with opinions similar to those of other reports; this refers to items such as chocolates, greasy foods, milk, eggs, and sugary drinks [6,8,13,14]. Eating peanuts, which are commonly called groundnuts across Nigeria, was the most frequently cited associated factor for acne in our study irrespective of the presence of acne. This was also reported by Aiyedun et al [28] in South West Nigeria. The widespread strong belief that intake of nuts in general will result in acne outbreaks has little evidence in support [1,13,16]. Groundnuts are a staple food in the Northern Nigeria and are consumed in a variety of ways; the corollary of this misperception in a resource-poor setting like ours is that adolescents may avoid this important and inexpensive source of protein used in a variety of snacks, soups, and meals, which is needed in a study population like ours, in which a sizable proportion of individuals (39%) had underweight. Any denial of nutrient-rich foods because of erroneous beliefs would further compromise growth and development in this age group [29].

Unlike Ražnatović Đurović et al [6], who found no difference in the perceptions of aggravating and ameliorating factors of acne between adolescents with and those without acne in Montenegro, we found some differences in perceptions surrounding acne between the 2 groups [6]. There was a higher proportion of individuals who linked acne with a biological phenomenon (puberty, maturity, menstruation, or genetic or familial factors) among participants with acne than in those without clinically diagnosed acne. On the contrary, perceptions that seem judgmental were expressed to a significantly higher extent by those without acne, such as associating the presence of acne with the use of skin lightening products, which is a practice largely disapproved of by society. The perception may not be completely unfounded as the use of topical steroids is associated with a form of acne and has become increasingly common in young persons in Nigeria [8,20].

The view that some adolescents have of persons with acne being filthy or unhygienic has been reported in other studies where the dark color of an open comedone caused by keratin oxidation was interpreted as dirt [1]. It has been demonstrated that these misconceptions add to the psychological burden of acne and are linked to obsessional face washing or persistent use of medicated soaps, which can irritate the skin [8,20].

The significantly lesser positive attitudes or perceptions held by people without acne (27.3% vs 72.7%; *P*=.04) depict how persons not affected by a particular condition may have less tolerant or favorable opinions of those with the condition. Other studies have reported that this attitude usually underlies poor social behaviors toward persons with acne, affecting mental health, which may be explored in adolescents with acne in Jos.

### Limitations

The study’s’ limitations include the possibility of recall and response bias. The data could also have been skewed by the higher proportion of female participants. The relationship between severity and the variables may be investigated for a better knowledge of the epidemiology of acne.

### Conclusions

Despite these limitations, our study suggests that acne is prevalent in adolescents in Jos, and there were a variety of misconceptions and unfavorable perceptions of the condition within this age group. This highlights the need for appropriate health education inculcated in school health programs, including services and referral by dermatologists. Additional research on the effects of acne on adolescents and their treatment-seeking behavior will also help to understand the burden of acne in our settings.

## Abbreviations

AOR: adjusted odds ratio
LGA: Local Government Area
OR: odds ratio
